# Comparison Study of Myocardial Radiomics Feature Properties on Energy-Integrating and Photon-Counting Detector CT

**DOI:** 10.3390/diagnostics12051294

**Published:** 2022-05-23

**Authors:** Isabelle Ayx, Hishan Tharmaseelan, Alexander Hertel, Dominik Nörenberg, Daniel Overhoff, Lukas T. Rotkopf, Philipp Riffel, Stefan O. Schoenberg, Matthias F. Froelich

**Affiliations:** 1 Department of Radiology and Nuclear Medicine, University Medical Center Mannheim, Heidelberg University, Theodor-Kutzer-Ufer 1-3, 68167 Mannheim, Germany; isabelle.ayx@medma.uni-heidelberg.de (I.A.); hishan.tharmaseelan@medma.uni-heidelberg.de (H.T.); alexander.hertel@medma.uni-heidelberg.de (A.H.); dominik.noerenberg@medma.uni-heidelberg.de (D.N.); daniel.overhoff@umm.de (D.O.); philipp.riffel@umm.de (P.R.); stefan.schoenberg@umm.de (S.O.S.); 2 Department of Diagnostic and Interventional Radiology and Neuroradiology, Bundeswehr Central Hospital Koblenz, Rübenacher Straße 170, 56072 Koblenz, Germany; 3 Department of Radiology, German Cancer Research Center, Im Neuenheimer Feld 280, 69120 Heidelberg, Germany; l.rotkopf@dkfz-heidelberg.de

**Keywords:** photon-counting computed tomography, feature stability, cardiac imaging, radiomics

## Abstract

The implementation of radiomics-based, quantitative imaging parameters is hampered by a lack of stability and standardization. Photon-counting computed tomography (PCCT), compared to energy-integrating computed tomography (EICT), does rely on a novel detector technology, promising better spatial resolution and contrast-to-noise ratio. However, its effect on radiomics feature properties is unknown. This work investigates this topic in myocardial imaging. In this retrospective, single-center IRB-approved study, the left ventricular myocardium was segmented on CT, and the radiomics features were extracted using pyradiomics. To compare features between scanners, a *t*-test for non-paired samples and F-test was performed, with a threshold of 0.05 set as a benchmark for significance. Feature correlations were calculated by the Pearson correlation coefficient, and visualization was performed with heatmaps. A total of 50 patients (56% male, mean age 56) were enrolled in this study, with equal proportions of PCCT and EICT. First-order features were, nearly, comparable between both groups. However, higher-order features showed a partially significant difference between PCCT and EICT. While first-order radiomics features of left ventricular myocardium show comparability between PCCT and EICT, detected differences of higher-order features may indicate a possible impact of improved spatial resolution, better detection of lower-energy photons, and a better signal-to-noise ratio on texture analysis on PCCT.

## 1. Introduction

Rising interest in the field of quantitative medical image analysis has developed over the past decade, driven by the promise of extracting additional information from pixel-based information from imaging data, often referred to as radiomics [1]. Radiomics describes a technique that enables the radiologist not only to count on the visual interpretation of the images but also to incorporate quantitative data into the diagnostic decision process. Radiomics features can be divided into several subtypes, starting with the group of first-order statistics, which describe the distribution of voxel intensities, without considering the spatial relationship. Higher-order statistics summarize texture features, shape-based parameters, and transform-based parameters. Texture features define the spatial distribution of voxels and, hence, visualize the heterogeneity of the area of interest [2]. Through radiomics analysis, many image characteristics not visible to the human eye can be extracted [1,3], using dedicated software packages [4]. These techniques have resonated, in particular, in the field of oncologic imaging and tumor analysis, with promising results in terms of tumor classification [5,6,7] and outcome prediction [8,9]. 

Recently, texture analysis has been, increasingly, applied in cardiac imaging, focusing mainly on cardiac MRI [10,11,12], however, first analyses have also been performed on cardiac CT [13,14]. Although there has been a decrease in mortality from cardiovascular disease (CVD) in recent years, this entity remains the number one cause of death worldwide, proving the importance of further diagnostic tools in this field [15]. The need for better cardiac diagnostic tools has been recognized by medical societies, resulting in the readjustment of guidelines and corresponding recommendations of cardiac CT in these patients [16]. Recently, this was further emphasized by a study showing a lower frequency of major procedure-related complications in patients with stable chest pain and intermediate pretest probability of coronary artery disease, who underwent initial CT instead of initial invasive coronary angiography [17]. 

In this context, quantitative radiomics features have shown a notable potential for better risk stratification, by quantification of coronary plaques [18] and perivascular fat [19]. Furthermore, first analyses did investigate the potential application of radiomics to myocardial fibrosis [20], in order to overcome the inferiority of cardiac CT, when compared to cardiac MRI. While this application is very promising, from a clinical point of view, the utilization of radiomics in everyday clinical care is severely hampered by a lack of feature stability: various parameters can influence the results from texture analysis, including different contrast media phases, slice thickness, and spatial resolution [21,22]. Especially, optimal spatial resolution and signal-to-noise ratio are known to be the two most important image quality factors, for accurate texture analysis [23,24,25]. This may be, partly, due to the current widespread use of conventional energy-integrating detectors (EID), which, indirectly, convert X-ray photons to electrical signals, with an additional intermediate scintillator-based step, resulting in potentially suboptimal data acquisition and image noise compared to photon-counting computed tomography (PCCT).

The implementation of PCCT has the potential to address this obstacle. In comparison to conventional EID, PCCT converts the X-ray photons directly into electric pulses, without the intermediate step of converting them to visible light. This revolutionary technology achieves a better spatial resolution, as well as a higher contrast-to-noise ratio and lower beam-hardening artifacts [26,27]. However, the applicability and comparability of quantitative radiomics analysis are yet to be investigated.

Therefore, the aim of this study is to investigate the properties of radiomics features extracted from the myocardium on a PCCT, compared to conventional energy-integrating computed tomography (EICT). 

## 2. Materials and Methods

For this retrospective single-center study, patients gated with clinically indicated electrocardiography (ECG) contrast-enhanced cardiac PCCT and matched cardiac EICT patients were enrolled between September 2021 and February 2022. In total, 50 patients (28 males, 22 females, mean age 56 years, range: 26–79 years) were selected, and 25 patients (10 males, 15 females, mean age 57 years, range: 41–79 years) were scanned on a dual-source EID CT scanner, whereas the other 25 patients (18 males, 7 females, mean age 56 years, range: 26–78 years) were examined using a first-generation whole-body dual-source photon-counting detector (PCD) CT system. Patients were excluded in case of stenosis degree ≥ 50% in any coronary artery as well as in case of coronary artery stent implantation. Additionally, patients were excluded in case of visible myocardial damage, to ensure a homogeneous myocardium as a basis for this study. Based on these criteria, 27 PCCT and 41 EICT patients were excluded. This retrospective study was approved by the institutional review board and local ethics committee (ID 2021-659). All investigations were conducted according to the Declaration of Helsinki. 

### 2.1. Chest CT Imaging

Twenty-five patients were scanned using a 2 × 192 slice 3rd generation dual-source CT scanner (SOMATOM Force, Siemens Healthcare GmbH, Forchheim, Germany), using tube voltages of 100 kV and automatic dose modulation, whereas the other 25 patients were scanned using the first-generation whole-body dual-source PCD CT system (NAEOTOM Alpha, Siemens Healthcare GmbH, Forchheim, Germany), using a prospective ECG gated sequential mode with a tube voltage of 120 kV and automatic dose modulation with a CARE keV IQ setting of 64. The primary, performed, and unenhanced scan was not included in the analysis of this study. In both CT scanner protocols, an optional application of ß-blocker (5–10 mg, Metoprolol, Recordati Pharma GmbH, Ulm, Germany), to lower the heart rate for reaching a target heart rate below 65 beats per minute, was followed by the application of sublingual nitroglycerin (0.8 mL). Via an antecubital vein, an iodinated contrast medium (70–80 mL Imeron 400, Bracco Imaging Deutschland GmbH, Konstanz, Germany), followed by a 20 mL saline chaser (NaCl 0.9%), was injected, applying a weight-based flow rate (5–6 mL/s). Bolus tracking was used to trigger the start of coronary CTA, by placing a region of interest (ROI) in the descending thoracic aorta (threshold 140 HU at 90 kV). 

### 2.2. Chest CT Imaging Analysis

Axial images of contrast-enhanced CCTA were reconstructed with a slice thickness of 0.6 mm (increment 0.4 mm PCCT, increment 0.3 mm EICT), using a soft vascular kernel (Bv40). For PCCT, this analysis is based on the non-spectral T3D image series. From this axial image dataset, short-axis view images were reformatted using a 5 mm slice thickness, according to published suggestions in the literature (11). All data were anonymized, exported, and stored in digital imaging and communications in a medicine (DICOM) file format, for further processing. The DICOM file format was converted to a NIFTI file format, for usability with the applied segmentation tool (3D Slicer, Version 4.11) [28]. The whole left ventricular myocardium, excluding the trabecular structure and papillary muscle, was manually segmented by a radiologist with nine years of experience in cardiovascular imaging. 

### 2.3. Radiomics Feature Extraction

Left ventricular myocardial segmentations were further analyzed by a dedicated radiomics analysis framework (pyradiomics, version 3.0.1) [4]. Settings for the analysis can be found in the supplemental material. The following feature types were extracted for each patient enrolled in the study: first-order, neighboring gray tone difference (NGTDM), gray level co-occurrence matrix (GLCM), gray level run length matrix (GLRLM), gray level size zone (GLSZM), and gray level dependence matrix (GLDM).

### 2.4. Statistical Analysis

All statistical analyses were performed in R for statistical analysis (version 4.1.2, R Foundation for Statistical Computing) [29]. For the analysis, the packages tableone, dplyr, ggplot2, ggcorrplot, and caret were utilized. To evaluate differences between groups, *t*-test for non-paired samples was applied, to compare means of quantitative variables. To evaluate a significant difference in variance, the F-test was performed on a variance of the standard deviation of each feature for each scanner, and a threshold of 0.05 was, likewise, set as a benchmark for significance. Fisher’s exact test was employed for categorical variables. A *p*-value of below 0.05 was assumed as statistically significant. Feature correlations were calculated by the Pearson correlation coefficient and visualized in unclustered heatmaps, for both patient groups. Then, hierarchical clustering was applied and additional clustered heatmaps were created. The mean and standard deviation of radiomics parameters were compared between both groups, as described above. Distributions were visualized as boxplots.

## 3. Results

### 3.1. Patient Collective

**Figure 1:**
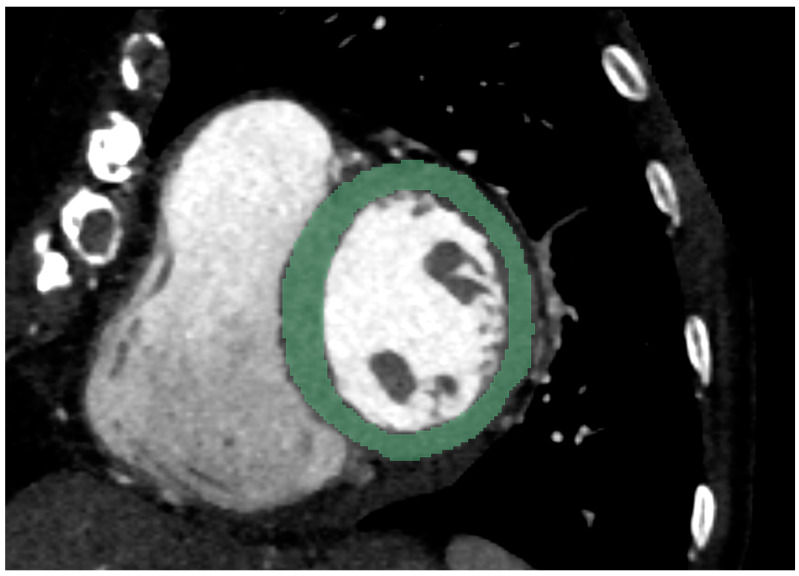
Segmentation of the left-ventricular myocardium was performed on short-axis views, with a slice thickness of 5 mm. An example case of a 79-year female on EICT is shown.

Based on inclusion criteria, a total of 50 CT scans of patients without significant coronary artery stenosis, defined as stenosis above 50% of vessel lumen narrowing, and without visual signs of myocardial damage, were enrolled in this study. In our study population, 44% were female and had a mean age of 56. The patient’s characteristics, as well as scan characteristics and manually measured mean HU values with SD, are summarized in Table 1. For these patients, segmentation of the left ventricular myocardium with the exclusion of papillary muscle and trabecular structures was performed, according to the approach presented in the Materials and Methods. Figure 1 shows an example segmentation of the left ventricular myocardium, in a short-axis view.

### 3.2. Cluster Analysis

**Figure 2:**
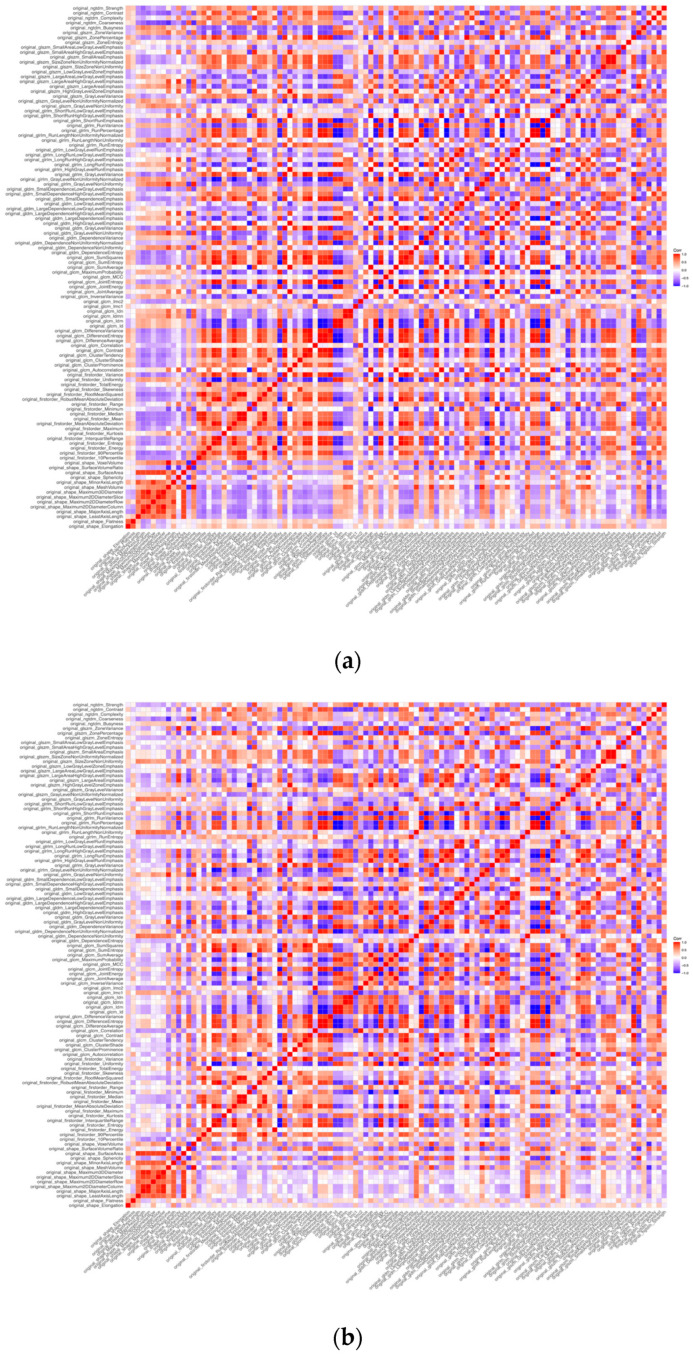
Correlation matrix of radiomics features extracted from myocardial segmentation. (**a**) Heatmap of myocardial radiomics features from energy-integrating CT (EICT) scans of 25 patients. (**b**) Heatmap of myocardial radiomics features from photon-counting CT (PCCT) scans of 25 patients.

An unclustered heatmap of radiomics features of the left ventricular myocardium, for all patients, was created after feature extraction and standardization for each scanner, respectively, showing a partly comparable distribution of feature correlations (Figure 2). 

**Figure 3:**
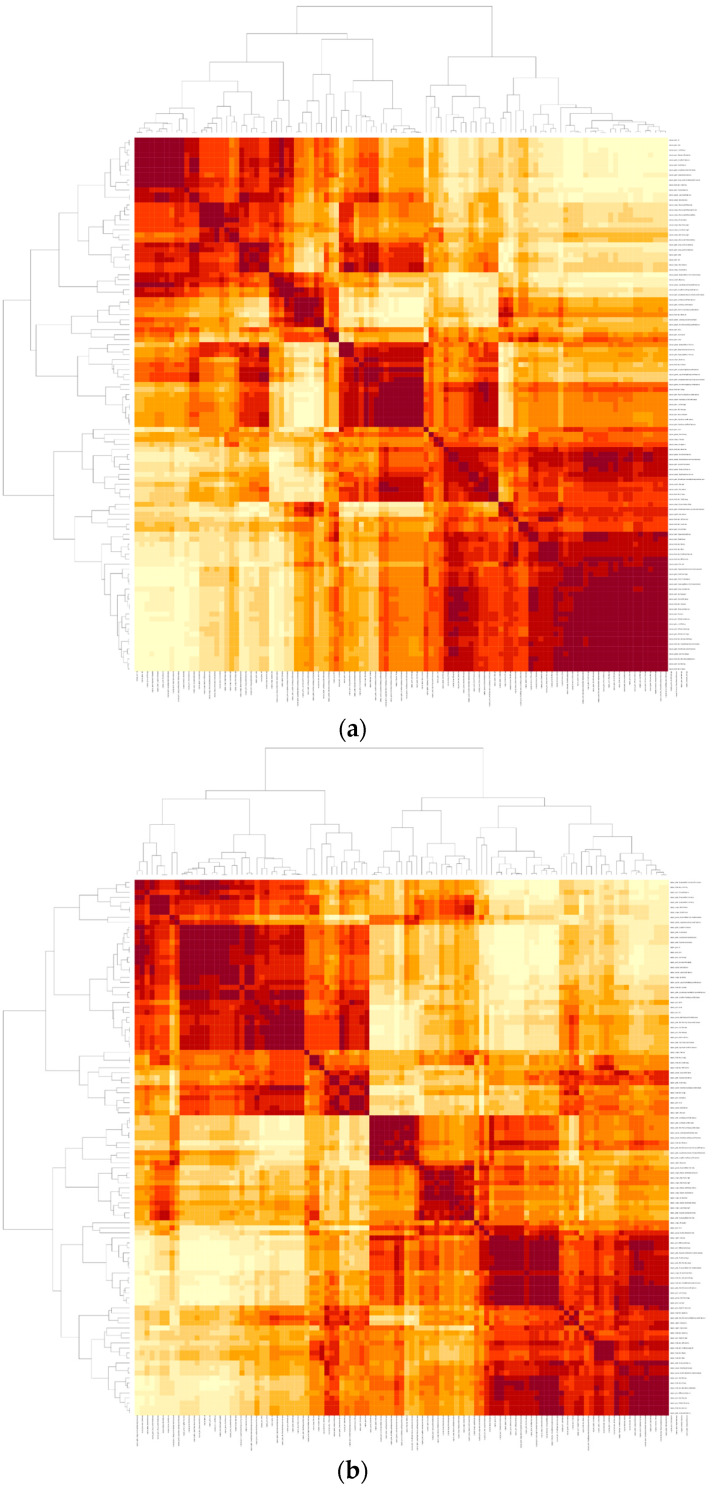
Unsupervised clustering of radiomics features. (**a**) Heatmap for energy-integrating CT (EICT). (**b**) Heatmap for photon-counting CT (PCCT).

Unsupervised hierarchical clustering of features was performed and is visualized as a heatmap for each scanner group, in Figure 3. 

### 3.3. Radiomics Feature Assessment

To assess comparative feature properties between both scanner collectives, the mean and the standard deviation for each feature were calculated. A significant difference between the mean of PCCT and EICT was detected in only two first-order radiomics features (firstorder_Maximum and firstorder_Skewness, Table 2), as well as in six features of Gray Level Co-occurrence Matrix (GLCM), one feature of Gray Level Dependence Matrix (GLDM), five features of Gray Level Size Zone Matrix (GLSZM) and two features of Neighbouring Grey Tone Difference Matrix (NGTDM). A detailed overview of those features is offered in Table 3. 

Only one first-order feature showed a significant difference of standard deviation among the two different scanners (firstoder_Median, Table 2). Significant differences in standard deviation were shown by six features of GLCM, three features of GLDM, five features of GLRLM, three features of GLSZM, and two features of NGTDM, as shown in Table 3. 

The features firstorder_mean and firstorder_median show a lower variation of measured mean and median Hounsfield attenuation in PCCT (firstorder_median SD EICT 25.88, PCCT 17.04, F-test 0.046, firstorder_mean SD EICT 26.86, PCCT 17.99, F-test 0.055). Apart from these two features, first-order features were similar between both scanners, indicating comparability of directly measured Hounsfield density values. 

With respect to the higher-order radiomics features, notable differences between PCCT and EICT were observed, outlined by a significantly higher mean of glszm_ZoneEntropy (EICT 6.82, PCCT 6.66, *t*-test < 0.001) or a lower mean of glzsm_SizeZoneNonUniformity (EICT 1215.28, PCCT 1622.90, *t*-test 0.012) and glszm_GrayLevelNonUniformity (EICT 304.54, PCCT 397.07, *t*-test 0.011) (Table 3). A significantly higher mean of the feature glszm_SmallAreaEmphasis was found in the PCCT data sample, in comparison to the EICT (EICT 0.053, PCCT 0.056, *t*-test 0.028). An overview table of all investigated radiomics parameters is shown in Supplemental Table S1. 

## 4. Discussion

This work presents a first in-human comparison of myocardial radiomics feature properties and distributions from both EICT and PCCT. In general, similar feature properties between both groups were detectable, however, there were notable differences regarding feature classes: first-order features showed comparable mean values and standard deviations between both groups, indicating comparability of directly measured Hounsfield units within the myocardium. However, higher-order features were more heterogeneous and did, partly, show significant differences in mean and standard deviation between both groups. These results indicate that while simple Hounsfield-based measures may be comparable between both groups, texture features may need further evaluation in PCCT. These differences may be, possibly, explained by the entirely different detector systems and their influence on the image reconstruction workflow, higher resolution, and better detection of lower-energy photons.

In 2017, Hinzpeter et al. demonstrated the feasibility of using texture analysis for differentiation between normal and acute infarcted myocardium in CT, with a good to excellent intra- and interreader agreement for all first and second-level features, at all slice thicknesses. Additionally, the most accurate results were obtained at a slice thickness of 5 mm in their study, laying the focus on the impact of slice thickness on the texture analysis of the myocardium [14]. In contrast, Zhao et al. showed a significant difference between 1.25 mm and 5 mm slice thickness, using a chest phantom. In their study 1.25 mm and 2.5 mm slice thicknesses were better suitable for volume, density means, density SD gray-level co-occurrence matrix (GLCM) energy, and homogeneity, compared to 5 mm slice thickness. The reconstruction algorithm showed that even its influence on the radiomics features, by the lung reconstruction algorithm (being best for density to mean), whereas the standard reconstruction algorithm was the best for density SD [30]. However, the time between application of the contrast agent and scan performance may influence texture features: Kim et al. demonstrated good stability of different texture features extracted from lung nodules, namely SD, variance, entropy, sphericity, discrete compactness, GLCM IDM, GLCM contrast, and GLCM entropy, between a scan delay of 30 and 180 s. However, texture features from pre-contrast CT scans differed, significantly, from those on contrast-enhanced scans [21]. A significant influence of the CT scanner manufacturer and acquisition parameters on a CT phantom was demonstrated by Mackin et al. in 2018. They investigated the stability of texture analysis parameters, by comparing 16 different CT scanners by four different manufacturers. The variability they observed between different CT scanners implied that the repeatability and, hence, quality of radiomics studies depends strongly on the consistency of image acquisition and reconstruction [31]. The effect of reconstruction algorithms on feature analysis, regarding lesion size, attenuation, and texture, was also demonstrated by Solomon et al. The radiation dose, however, affected mainly the estimation of lesion size, conspicuity, and intralesional pixel value distribution feature, with only a little effect on lesion texture analysis [32]. 

Summarizing not only our results but also the literature, radiologists must be aware of the fact that differences in texture analysis can have multiple reasons—an actual texture change is only one of them. Therefore, our first-in-human feasibility study must be interpreted in the context of the following limitations. The study presented is retrospective, was performed at a single-center, and does include a limited number of patients. However, this is due to the limited number of patients scanned with PCCT as an emerging technology and the strict patient selection criteria, in terms of myocardial disease, which did lead to a significant number of excluded patients. Unfortunately, gender differs significantly between both groups, so further analysis in the future should focus on a more homogeneous patient population. Moreover, the study does not include a dedicated feature stability analysis in consecutive patients but consists of different patients in both groups—a result of the retrospective character not allowing additional CT scans, which are not clinically indicated. Such an analysis would, therefore, be possible in a phantom study. Additionally, a significant limitation is the different tube voltage between PCCT and EICT, however, until now a modulation of tube voltage in the PCCT scanner used in this study is not possible for the chosen protocol. Apart from that, reconstruction parameters were kept as constant as possible between both groups. The same reconstruction kernel and slice thickness were used. 

In conclusion, this study investigates radiomics feature properties in EICT and PCCT on homogeneous human myocardium in a retrospective, group-comparison approach, to evaluate feature stability and properties. This study is, explicitly, not focused on pathology differentiation, but rather it is intended as a basis for future work in this direction. While first-order features showed comparable values between both groups, higher-order radiomics features seem to be more heterogeneous and may require a dedicated readjustment for PCCT. This may be due to inherent features of the PCCT technology, including improved spatial resolution, better detection of lower-energy photons, and better signal-to-noise ratio. Prospectively, extraction of multi-energy properties from the now-acquired quantum datasets may help to understand texture-feature variability better and to address problems of heterogeneity.

## Figures and Tables

**Table 1:** Patient collective overview. Mean and (SD) given for continuous variables.

	EICT	PCCT	*p* Value
Patient parameters			
*n*	25	25	
Age	56.88 (10.79)	56.08 (13.98)	0.822
Sex	10 male (40.0%)	18 male (72.0%)	0.046
Stent	0	0	N/A
Significant stenosis (>50%)	0	0	N/A
Agatston Score	55.38 (110.55)	38.29 (87.76)	0.548
Mean HU Value	114.52 (50.87)	125.13 (42.04)	0.123
Scanner parameters			
Tube voltage	100	120	N/A
Slice thickness	5 mm	5 mm	N/A
Kernel	Bv40	Bv40	N/A
Tube	Vectron ^®^	Vectron ^®^	N/A

**Table 2:** Comparison of first-order radiomics parameters, extracted from myocardial segmentation. Variables mit significant differences in groups shown, for full table refer to supplemental material.

Feature Mean (SD)	EICT	PCCT	*t* Test	F Test
*n*	25	25		
First order features				
original_firstorder_Maximum	517.90 (120.68)	604.00 (119.59)	0.015	0.965
original_firstorder_Median	124.93 (25.88)	126.65 (17.04)	0.782	0.046
original_firstorder_Skewness	−0.28 (0.97)	0.28 (0.86)	0.035	0.557

**Table 3:** Higher order radiomics features, with significant differences in mean and/or SD.

Feature Mean (SD)	EICT	PCCT	*t* Test	F Test
*n*	25	25		
Gray Level Co-Occurrence Matrix (GLCM)				
original_glcm_Contrast	2.76 (1.00)	3.43 (1.30)	0.049	0.206
original_glcm_Correlation	0.64 (0.03)	0.57 (0.07)	<0.001	<0.001
original_glcm_Idmn	1.00 (0.0006)	1.00 (0.001)	0.115	<0.001
original_glcm_Idn	0.97 (0.00)	0.97 (0.01)	0.194	<0.001
original_glcm_Imc1	−0.15 (0.02)	−0.13 (0.04)	0.002	0.007
original_glcm_Imc2	0.73 (0.04)	0.66 (0.08)	0.001	<0.001
original_glcm_InverseVariance	0.46 (0.01)	0.45 (0.02)	0.016	0.062
original_glcm_MCC	0.71 (0.04)	0.67 (0.07)	0.025	0.011
Gray Level Dependence Matrix (GLDM)				
original_gldm_DependenceNonUniformityNormalized	0.07 (0.01)	0.08 (0.01)	0.074	0.042
original_gldm_LowGrayLevelEmphasis	0.00 (0.00)	0.00 (0.00)	0.074	<0.001
original_gldm_SmallDependenceLowGrayLevelEmphasis	0.00 (0.00)	0.00 (0.00)	0.032	<0.001
Gray Level Run Length Matrix (GLRLM)				
original_glrlm_LongRunEmphasis	2.87 (0.60)	2.80 (0.93)	0.772	0.04
original_glrlm_LongRunLowGrayLevelEmphasis	0.01 (0.00)	0.01 (0.01)	0.202	0.016
original_glrlm_LowGrayLevelRunEmphasis	0.00 (0.00)	0.00 (0.00)	0.076	<0.001
original_glrlm_RunVariance	0.82 (0.28)	0.79 (0.44)	0.802	0.033
original_glrlm_ShortRunLowGrayLevelEmphasis	0.00 (0.00)	0.00 (0.00)	0.062	<0.001
Gray Level Size Zone Matrix (GLSZM)				
original_glszm_GrayLevelNonUniformity	304.54 (69.82)	397.07 (160.54)	0.011	<0.001
original_glszm_LowGrayLevelZoneEmphasis	0.00 (0.00)	0.01 (0.00)	0.111	<0.001
original_glszm_SizeZoneNonUniformity	1215.38 (435.47)	1622.80 (651.64)	0.012	0.054
original_glszm_SizeZoneNonUniformityNormalized	0.27 (0.04)	0.29 (0.03)	0.023	0.662
original_glszm_SmallAreaEmphasis	0.53 (0.04)	0.56 (0.03)	0.028	0.488
original_glszm_SmallAreaLowGrayLevelEmphasis	0.00 (0.00)	0.00 (0.00)	0.068	<0.001
original_glszm_ZoneEntropy	6.82 (0.11)	6.66 (0.17)	<0.001	0.054
Neighbouring Grey Tone Difference Matrix (NGTDM)				
original_ngtdm_Busyness	6.54 (2.31)	9.96 (7.85)	0.042	<0.001
original_ngtdm_Coarseness	0.00 (0.00)	0.00 (0.00)	0.011	0.178
original_ngtdm_Contrast	0.01 (0.00)	0.01 (0.00)	0.153	0.013

## Data Availability

The data presented in this study are available on request from the corresponding author.

## Supplementary Materials

The following supporting information can be downloaded at: https://www.mdpi.com/article/10.3390/diagnostics12051294/s1, Table S1: overview of all parameters; supplemental information.
