# Supplementary material for: Comparison Study of Myocardial Radiomics Feature Properties on Energy-Integrating and Photon-Counting Detector CT
Source: Diagnostics (Basel). 2022 May 23;12(5):1294. doi: 10.3390/diagnostics12051294 (PMC9141463; doi:10.3390/diagnostics12051294)
Supplement: Supplementary file 1 [file diagnostics-12-01294-s001.zip › diagnostics-1712230-supplementary.pdf]

Supplemental Material

Supplemental Table S1. Overview of all parameters

Feature Mean (SD)	EICT	PCCT	*t* test	F test
n	25	25		
First order Features				
original_firstorder_10Percentile	71.21 (20.17)	73.32 (14.39)	0.673	0.105
original_firstorder_90Percentile	179.70 (35.23)	183.56 (26.71)	0.664	0.183
original_firstorder_Energy	1353081205.22 (431837986.34)	1564414365.82 (428476193.07)	0.089	0.970
original_firstorder_Entropy	2.94 (0.28)	2.96 (0.28)	0.866	0.971
original_firstorder_InterquartileRange	52.51 (10.58)	54.91 (11.08)	0.436	0.821
original_firstorder_Kurtosis	11.78 (5.02)	12.12 (5.59)	0.824	0.603
original_firstorder_Maximum	517.90 (120.68)	604.00 (119.59)	0.015	0.965
original_firstorder_MeanAbsoluteDeviation	36.42 (7.35)	37.13 (7.40)	0.734	0.970
original_firstorder_Mean	125.40 (26.86)	128.42 (17.99)	0.642	0.055
original_firstorder_Median	124.93 (25.88)	126.65 (17.04)	0.782	0.046
original_firstorder_Minimum	-371.27 (112.63)	-326.57 (119.02)	0.179	0.789
original_firstorder_Range	889.18 (183.37)	930.57 (173.77)	0.417	0.795
original_firstorder_RobustMeanAbsoluteDeviation	22.14 (4.45)	23.15 (4.72)	0.441	0.770
original_firstorder_RootMeanSquared	136.35 (28.12)	139.42 (19.43)	0.655	0.076
original_firstorder_Skewness	-0.28 (0.97)	0.28 (0.86)	0.035	0.557
original_firstorder_TotalEnergy	1353081205.22 (431837986.34)	1564414365.82 (428476193.07)	0.089	0.970
original_firstorder_Uniformity	0.17 (0.03)	0.17 (0.03)	0.663	0.841
original_firstorder_Variance	2932.46 (1195.57)	2998.05 (1177.88)	0.846	0.942
Gray Level Co-Occurrence Matrix (GLCM)				
original_glcm_Autocorrelation	458.81 (219.23)	389.94 (182.53)	0.233	0.376
original_glcm_ClusterProminence	1948.87 (1356.41)	1960.21 (1495.19)	0.978	0.637
original_glcm_ClusterShade	-2.40 (49.66)	21.11 (40.77)	0.074	0.340
original_glcm_ClusterTendency	12.91 (5.03)	12.99 (5.08)	0.957	0.963
original_glcm_Contrast	2.76 (1.00)	3.43 (1.30)	0.049	0.206
original_glcm_Correlation	0.64 (0.03)	0.57 (0.07)	<0.001	<0.001

original_glcm_DifferenceAverage	1.14 (0.22)	1.28 (0.28)	0.057	0.233
original_glcm_DifferenceEntropy	1.94 (0.22)	2.05 (0.25)	0.112	0.463
original_glcm_DifferenceVariance	1.38 (0.50)	1.67 (0.62)	0.082	0.270
original_glcm_Id	0.60 (0.04)	0.58 (0.06)	0.078	0.280
original_glcm_Idm	0.57 (0.05)	0.53 (0.07)	0.074	0.267
original_glcm_Idmn	1.00 (0.00)	1.00 (0.00)	0.115	<0.001
original_glcm_Idn	0.97 (0.00)	0.97 (0.01)	0.194	<0.001
original_glcm_Imc1	-0.15 (0.02)	-0.13 (0.04)	0.002	0.007
original_glcm_Imc2	0.73 (0.04)	0.66 (0.08)	0.001	<0.001
original_glcm_InverseVariance	0.46 (0.01)	0.45 (0.02)	0.016	0.062
original_glcm_JointAverage	20.92 (4.38)	19.14 (4.71)	0.171	0.725
original_glcm_JointEnergy	0.05 (0.02)	0.04 (0.02)	0.320	0.816
original_glcm_JointEntropy	5.24 (0.53)	5.39 (0.57)	0.363	0.672
original_glcm_MCC	0.71 (0.04)	0.67 (0.07)	0.025	0.011
original_glcm_MaximumProbability	0.10 (0.03)	0.09 (0.04)	0.238	0.751
original_glcm_SumAverage	41.85 (8.76)	38.27 (9.42)	0.171	0.725
original_glcm_SumEntropy	3.68 (0.28)	3.68 (0.29)	0.980	0.877
original_glcm_SumSquares	3.92 (1.50)	4.10 (1.57)	0.671	0.827
Gray Level Dependence Matrix (GLDM)				
original_gldm_DependenceEntropy	6.72 (0.14)	6.66 (0.16)	0.163	0.598
original_gldm_DependenceNonUniformity	5187.88 (1504.68)	6089.68 (1706.31)	0.053	0.543
original_gldm_DependenceNonUniformityNormalized	0.07 (0.01)	0.08 (0.01)	0.074	0.042
original_gldm_DependenceVariance	17.60 (4.24)	15.70 (5.90)	0.197	0.112
original_gldm_GrayLevelNonUniformity	13444.88 (5984.33)	14296.93 (5930.22)	0.615	0.965
original_gldm_GrayLevelVariance	4.77 (1.91)	4.88 (1.88)	0.846	0.945
original_gldm_HighGrayLevelEmphasis	460.63 (219.06)	392.29 (181.92)	0.236	0.369
original_gldm_LargeDependenceEmphasis	82.29 (24.63)	74.29 (33.83)	0.344	0.127
original_gldm_LargeDependenceHighGrayLevelEmphasis	37458.58 (23387.03)	33225.80 (32027.68)	0.596	0.130

original_gldm_LargeDependenceLowGrayLevelEmphasis	0.22 (0.12)	0.23 (0.12)	0.723	0.907
original_gldm_LowGrayLevelEmphasis	0.00 (0.00)	0.00 (0.00)	0.074	<0.001
original_gldm_SmallDependenceEmphasis	0.07 (0.02)	0.08 (0.03)	0.140	0.297
original_gldm_SmallDependenceHighGrayLevelEmphasis	34.15 (17.62)	31.15 (12.39)	0.490	0.091
original_gldm_SmallDependenceLowGrayLevelEmphasis	0.00 (0.00)	0.00 (0.00)	0.032	<0.001
Gray Level Run Length Matrix (GLRLM)				
original_glrlm_GrayLevelNonUniformity	8742.78 (3359.73)	9619.14 (3251.00)	0.353	0.873
original_glrlm_GrayLevelNonUniformityNormalized	0.16 (0.03)	0.15 (0.03)	0.876	0.879
original_glrlm_GrayLevelVariance	5.73 (2.18)	5.75 (2.09)	0.979	0.848
original_glrlm_HighGrayLevelRunEmphasis	461.80 (219.17)	393.66 (182.01)	0.238	0.369
original_glrlm_LongRunEmphasis	2.87 (0.60)	2.80 (0.93)	0.772	0.040
original_glrlm_LongRunHighGrayLevelEmphasis	1320.16 (786.35)	1208.30 (994.76)	0.661	0.257
original_glrlm_LongRunLowGrayLevelEmphasis	0.01 (0.00)	0.01 (0.01)	0.202	0.016
original_glrlm_LowGrayLevelRunEmphasis	0.00 (0.00)	0.00 (0.00)	0.076	<0.001
original_glrlm_RunEntropy	4.19 (0.13)	4.13 (0.15)	0.113	0.436
original_glrlm_RunLengthNonUniformity	33063.86 (9100.95)	38416.38 (10688.76)	0.063	0.437
original_glrlm_RunLengthNonUniformityNormalized	0.60 (0.06)	0.62 (0.07)	0.189	0.170
original_glrlm_RunPercentage	0.73 (0.05)	0.75 (0.06)	0.257	0.140
original_glrlm_RunVariance	0.82 (0.28)	0.79 (0.44)	0.802	0.033
original_glrlm_ShortRunEmphasis	0.80 (0.04)	0.81 (0.05)	0.272	0.175
original_glrlm_ShortRunHighGrayLevelEmphasis	368.71 (170.44)	313.73 (127.68)	0.203	0.164
original_glrlm_ShortRunLowGrayLevelEmphasis	0.00 (0.00)	0.00 (0.00)	0.062	<0.001
Gray Level Size Zone Matrix (GLSZM)				
original_glszm_GrayLevelNonUniformity	304.54 (69.82)	397.07 (160.54)	0.011	<0.001

original_glszm_GrayLevelNonUniformityNormalized	0.07 (0.01)	0.07 (0.01)	0.618	0.724
original_glszm_GrayLevelVariance	23.71 (6.99)	22.84 (7.14)	0.665	0.922
original_glszm_HighGrayLevelZoneEmphasis	493.02 (228.33)	429.22 (181.95)	0.280	0.273
original_glszm_LargeAreaEmphasis	225617.74 (197772.52)	237076.43 (263655.32)	0.863	0.166
original_glszm_LargeAreaHighGrayLevelEmphasis	117066058.77 (185145181.54)	121442209.86 (195707701.69)	0.936	0.788
original_glszm_LargeAreaLowGrayLevelEmphasis	566.44 (600.65)	619.16 (609.19)	0.759	0.945
original_glszm_LowGrayLevelZoneEmphasis	0.00 (0.00)	0.01 (0.00)	0.111	<0.001
original_glszm_SizeZoneNonUniformity	1215.38 (435.47)	1622.80 (651.64)	0.012	0.054
original_glszm_SizeZoneNonUniformityNormalized	0.27 (0.04)	0.29 (0.03)	0.023	0.662
original_glszm_SmallAreaEmphasis	0.53 (0.04)	0.56 (0.03)	0.028	0.488
original_glszm_SmallAreaHighGrayLevelEmphasis	269.49 (131.57)	243.84 (100.07)	0.442	0.187
original_glszm_SmallAreaLowGrayLevelEmphasis	0.00 (0.00)	0.00 (0.00)	0.068	<0.001
original_glszm_ZoneEntropy	6.82 (0.11)	6.66 (0.17)	<0.001	0.054
original_glszm_ZonePercentage	0.06 (0.02)	0.07 (0.03)	0.229	0.359
original_glszm_ZoneVariance	225224.24 (197482.13)	236696.23 (263197.67)	0.862	0.167
Neighbouring Grey Tone Difference Matrix (NGTDM)				
original_ngtdm_Busyness	6.54 (2.31)	9.96 (7.85)	0.042	<0.001
original_ngtdm_Coarseness	0.00 (0.00)	0.00 (0.00)	0.011	0.178
original_ngtdm_Complexity	514.03 (251.11)	598.01 (200.18)	0.197	0.274
original_ngtdm_Contrast	0.01 (0.00)	0.01 (0.00)	0.153	0.013
original_ngtdm_Strength	0.16 (0.06)	0.15 (0.08)	0.462	0.403

Supplemental Information: pyradiomics settings. Extraction parameters{'minimumROIDimensions': 2, 'minimumROISize': None, 'normalize': False, 'normalizeScale': 1, 'removeOutliers': None, 'resampledPixelSpacing': None, 'interpolator': 'sitkBSpline', 'preCrop': False, 'padDistance': 5, 'distances': [1], 'force2D': False, 'force2Ddimension': 0, 'resegmentRange': None, 'label': 1, 'additionalInfo': True} Enabled filters: {'Original': {}} Enabled features: {'firstorder': [], 'glcm': [], 'gldm': [], 'glrlm': [], 'glszm': [], 'ngtdm': []}
